# Naturalist's Monthly Report

**Published:** 1811-12

**Authors:** 


### NATURALIST'S MONTHLY REPORT.

### OCTOBER.

> The fading, many-coloured woods,
> Shade deepening under shade, the country round
> Embrown.

THE wind has been more or less westerly during nearly the whole of the month. On the 3d and 4th it was south-east; on the 14th, 15th, and 17th, southerly; on the 27th, south-east; and on the 28th, first north, but afterwards south.

I have scarcely any recollection of more boisterous weather in a given number of days, than we have had in the course of this month. There were strong gales on the 1st, 4th, 5th, 7th, 12th, 25th, and 30th; fresh gales on the 3d, 6th, 8th, 11th, 13th, 18th, 20th, 22d, 24th, 27th, and 29th; and squally weather on the 2d, 25th, and 26th.

The only days on which we had no rain were the 6th, 8th, 13th, 15th, 16th, 17th, 18th, 19th, and 23d; and the 17th was the only fine day in the course of the month.

October 1st. There was a thunder-storm this morning, but it was of short continuance.

Honey is this year in considerable abundance, owing, no doubt, in a great measure, to the fine hot weather which was prevalent during the principal part of the month of September. Its price is now considerably less than one-third of what it was this time last year. In the fine afternoons I have seen the bees returning laden from the heaths in such numbers bers, as to appear almost as though they were young swarms leaving their hive.

October 2. The leaves of the elm, and of several species of willow, fall.

October 4. The leaves of the sumach turn red and fall.

The pewits begin to collect in large flocks in the fields.

October 7. Owing to the late rains the rivers and brooks begin to overflow their banks. It is about this season that the eels are supposed to commence their migration towards the sea, and during the first autumnal floods they are generally caught in immense quantities at the mills and wiers, but as yet very few have been seen.

The winter crops of potatoes are dug up.

October 9th. House-flies begin to appear torpid.

October 11th. During the high wind the rooks dash about and play in a more sportive manner than such heavy birds would seem capable of. They have very evidently great delight in this kind of stormy weather.

October 14th. A woodcock which was shot this day is the first that I have heard of this season.

October 16th. The swallows and martins have taken their leave of us for the present year.

October 17th. The ivy is now in full flower; and flies of various species swarm about the blossoms.—Michaelmas peaches are ripe.

The upper leaves of the poplars, and the leaves of the weeping willow, the mulberry, some of the pollard ashes, and sallows, are yet left. Those of the elm and lime trees are quite gone.

October 18th. Mushrooms, which, a little while ago, were found in great abundance, are again become scarce.

It is a singular fact that several chafers (*scarabæus melolontha*) have, at different times lately, been seen in flight. When caught they appear to be very languid and weak.

October 20th. The Royston crows are returned.

October 23d. The fruit of the elder, barberry, black thorn, woodbine, holly, hedge-rose, spindle-tree (*Evonymus Europæus*), black briony (*tamus communis*), woody nightshade (*solanum dulcamara*), and dogberry (*cornus sanguinia*), is now ripe.

Starlings begin to collect together in large flocks; and the linnets and other small birds also congregate.

October 28th. Fieldfares are seen. The leaves of the hawthorn are quite gone.

October 31st. I scarcely recollect to have seen the gossamer floating in the course of the whole autumn. The wet weather has prevented it.

In consequence of the continued rain very few of the farmers have been yet able to sow their wheat. The summer fallows are completely drenched with wet.

The crops of acorns and beech mast, like those of the hazel nuts and walnuts, have in this neighbourhood almost wholly failed.

*Hampshire.*

